# Predictive models for non-response to conventional treatment in polymyositis and dermatomyositis

**DOI:** 10.3389/fmed.2025.1596858

**Published:** 2025-09-26

**Authors:** Bo Zang, Lishan Xu, Qixuan Liu, Yuan Yao, Hua Li, Dacheng Li, Lingwei Liu, Ruiwen Liang, Xinyue Zhao, Peng Zhao, Chunli Xu, Bin Liu

**Affiliations:** ^1^Department of Rheumatology and Immunology, The Affiliated Hospital of Qingdao University, Qingdao, Shandong, China; ^2^Department of Rheumatology and Immunology, Liaocheng People's Hospital, Liaocheng, Shandong, China; ^3^Graduate Group of Epidemiology, University of California, Davis, Davis, CA, United States; ^4^Department of Nuclear Medicine, The Affiliated Hospital of Qingdao University, Qingdao, Shandong, China; ^5^Department of Geriatrics, Shengli Oilfield Central Hospital, Dongying, Shandong, China; ^6^Department of Pathology, The Affiliated Hospital of Qingdao University, Qingdao, Shandong, China; ^7^Department of Neurology, The Affiliated Hospital of Qingdao University, Qingdao, Shandong, China

**Keywords:** idiopathic inflammatory myopathies, FDG-PET/CT, muscle biopsies, predictive model, personalized treatment

## Abstract

**Introduction:**

Polymyositis (PM) and dermatomyositis (DM) are the major subtypes of idiopathic inflammatory myopathies with heterogeneous treatment responses. This study aimed to develop a predictive model for identifying PM/DM patients who are non-responsive to conventional therapy by assessing a range of diagnostic tools to guide individualized treatment.

**Methods:**

Patients with PM/DM from two medical centers (January 2010-December 2024) were included. Baseline and 12-week post-treatment clinical data were collected. Logistic regression was employed to develop both comprehensive and non-invasive predictive models. Model performance was validated using the area under the receiver operating characteristic curve (AUC) in internal and external test sets.

**Results:**

The Qingdao cohort comprised 84 patients (age 57.7 ± 1.5 years; 54 females [62.5%]; DM = 53, PM = 31) and the Liaocheng cohort included 22 patients (age 56.4 ± 3.8 years; 15 females [68.2%]; DM = 13, PM = 9). Gender, mSUVmax, and muscle bundle atrophy were identified as independent predictive factors. The comprehensive model achieved an AUC of 0.900 (95% confidence intervals [CI]: 0.813-1.009) in the training set, demonstrating superior diagnostic performance compared to the non-invasive model. The non-invasive model yielded AUCs of 0.857 (95% CI: 0.766-0.972), 0.742 (95% CI: 0.599-0.984), and 0.765 (95% CI: 0.632-0.987) in the training, internal test, and external test sets, respectively, indicating broader applicability across different cohorts. Both models showed good discrimination and calibration, and decision curve analysis further confirmed their clinical value.

**Discussion:**

These findings suggest that predictive models based on clinical, pathological, and imaging features can effectively identify PM/DM patients who are non-responsive to conventional therapy, potentially providing a tool for personalized treatment.

## 1 Introduction

Idiopathic inflammatory myopathies (IIM) represent a heterogeneous group of autoimmune diseases that primarily affect skeletal muscles, encompassing polymyositis (PM), dermatomyositis (DM), antisynthetase syndrome, immune-mediated necrotizing myopathy, inclusion body myositis, and overlap myositis (1). Recent advancements in diagnostic and pathogenetic understanding have revealed an increasing disease burden, with epidemiological studies reporting incidence rates of 0.2–2.0 cases per 100,000 person-years and prevalence estimates ranging from 2 to 25 cases per 100,000 people (2). The clinical spectrum of IIM extends beyond characteristic proximal muscle weakness to include multisystem involvement, manifesting as pathognomonic skin eruptions (particularly in DM), interstitial lung disease (ILD), arthralgia, and arrhythmias (3). Among these subtypes, DM demonstrates the highest prevalence at 6–7 cases per 100,000 person-years, followed by PM as the second most common clinical entity Significant inter-subtype variation exists in phenotypic presentation, therapeutic approaches, and long-term outcomes (4).

A large Swedish cohort study has revealed that PM/DM mortality is significantly higher than that of the general population, particularly in the first-year post-diagnosis (5). Conventional treatment typically involves corticosteroids, often combined with one or more immunosuppressive agents to minimize steroid-related side effects (6). However, a substantial proportion of patients exhibit inadequate responses to conventional therapy (7). In cases where patients demonstrate non- response to conventional therapy, it is essential to consider the accuracy of the initial diagnosis, the adequacy of drug dosing, or the overly rapid tapering of drug treatment. Advances in PM/DM pathogenesis have facilitated the development of novel therapeutics. Clinical trials have indicated that intravenous immunoglobulin (IVIG) effectively reduced disease activity in DM, while emerging therapies including the neonatal Fc receptors inhibitors, type I interferon inhibitors, and engineered T-cell therapies, have demonstrated significant potential for clinical application (8–10).

This study developed a nomogram model that combines 18F-fluorodeoxyglucose positron emission tomography/computed tomography (18F-FDG PET/CT) imaging features, muscle biopsy findings, and clinical factors to predict the prognosis of PM/DM patients after conventional treatment. The model is designed to facilitate the early identification of high-risk patients, enabling timely adjustments in treatment strategies to improve clinical outcomes.

## 2 Materials and methods

### 2.1 Patients

**Figure 1:**
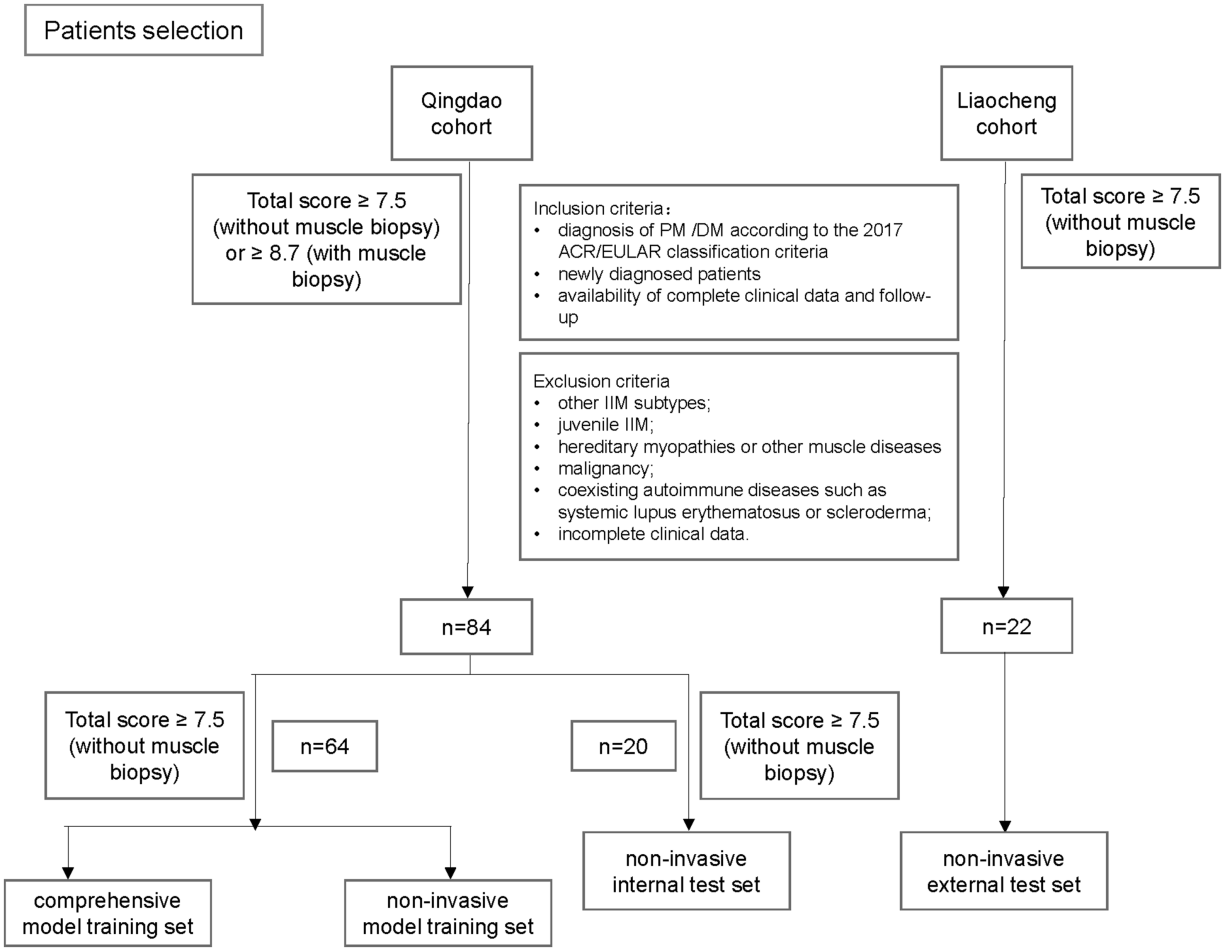
Flow diagram of patient selection from two medical centers PM, polymyositis; DM, dermatomyositis; IIM, idiopathic inflammatory myopathies.

This study analyzed PM/DM patients treated at the Affiliated Hospital of Qingdao University and Liaocheng People’s Hospital between January 2010 and December 2024. The inclusion criteria were: (1) diagnosis of PM or DM according to the European League Against Rheumatism (EULAR)/American College of Rheumatology (ACR) classification criteria (11); (2) newly diagnosed patients; (3) availability of complete clinical data and follow-up; and (4) patients were required to have pre-treatment magnetic resonance imaging (MRI) and PET/CT imaging. Exclusion criteria included: (1) other IIM subtypes; (2) juvenile IIM; (3) hereditary myopathies or other muscle diseases; (4) malignancy; (5) coexisting autoimmune diseases such as systemic lupus erythematosus or scleroderma; and (6) incomplete clinical data. Patients were divided into development and validation cohorts, with the Qingdao cohort split into training and internal test sets, while the Liaocheng cohort was used for external validation. The patient selection flowchart is shown in Figure 1. Conventional treatment consisted of corticosteroids combined with one immunosuppressive agent (azathioprine 2 mg/kg/day, methotrexate 15–20 mg/week, or cyclophosphamide 0.1 qod). The clinical assessment of patients was evaluated using the six core set measures proposed by ACR/EULAR in 2016: (1) Patient global assessment; (2) Physician global assessment; (3) Manual muscle testing; (4) Health Assessment Questionnaire; (5) Extra-muscular disease activity assessment; and (6) Serum creatine kinase (CK) levels. The total improvement score (TIS, 0–100) was the sum of individual Core set measures scores, with “good treatment response” defined as a TIS ≥ 20 after 12 weeks of conventional therapy while a TIS < 20 was considered indicative of non-response to conventional treatment (12). The study adhered to the principles of the Helsinki Declaration and was approved by the Ethics Committee of Qingdao University Affiliated Hospital (QYFY WZLL 27891), with a waiver of informed consent for this retrospective design.

### 2.2 Autoantibodies

Myositis-specific autoantibodies (MSAs), including anti-Jo-1, anti-MDA5, anti-TIF1γ, anti-Mi-2, anti-SAE, anti-NXP-2, and anti-EJ, as well as myositis-associated autoantibodies (MAAs), such as anti-PM-Scl, anti-RNP, and anti-Ro/SSA autoantibodies were detected using a western blot assay (EUROIMMUN, Germany).

### 2.3 EMG

Electromyography (EMG) was performed in a non-sedated state using needle electrodes to record electrical activity from multiple proximal muscles (deltoid, biceps, quadriceps, gastrocnemius, etc.). The spontaneous activity, including fibrillation potentials (Fibs) and positive sharp waves (PSWs), was assessed for frequency and distribution. “Abundant spontaneous potentials (SPs)” was defined as a frequency ≥2 per second of Fibs/PSWs across multiple muscle sites. The testing was conducted by experienced electrophysiologists, with independent review by two experts to minimize subjective bias, and discrepancies were resolved through consensus.

### 2.4 MRI

Muscle MRI was conducted using 3.0 T Intera (GE Signa HDX, United States) and Siemens SKYRA (Japan) scanners. Imaging data were subsequently subjected to independent evaluation by two radiologists with extensive experience in the field of musculoskeletal diseases, following a double-blind protocol. Abnormal skeletal muscle signals were identified on T2-weighted images, with long TE fat-saturation or short tau inversion recovery sequences. Muscle edema, atrophy, fascial edema, and subcutaneous/soft tissue edema were graded on a 4-point scale (0-normal, 1-mild, 2-moderate, 3-severe) for each muscle group (gluteal, adductors, hamstrings, and quadriceps), yielding total scores from 0 to 12. The final score was the mean of both radiologists’ assessments.

### 2.5 PET-CT imaging

Patients fasted for 6 h, with blood glucose levels < 11.1 mmol/L (predominantly < 8.3 mmol/L), followed by intravenous injection of 5.5–6.6 MBq/kg 18F-FDG (Pet, Beijing, China). Imaging was performed using the Discovery system (GE Discovery VCT, United States). Scans were independently assessed by two experienced nuclear medicine physicians, blinded to clinical information. Muscle FDG uptake was quantified using the maximum standardized uptake value (mSUVmax) method, as described by Matuszak et al. mSUVmax was calculated as the average of the 16 highest standardized uptake values from bilateral muscle groups (pectoralis major, deltoid, triceps brachii, biceps brachii, psoas major, gluteus, quadriceps, and adductors), normalized to the mean liver SUV (13). Measurements were taken at the muscle mid-point, excluding tendons, when FDG uptake was evenly distributed. Each physician performed two measurements, and the final value was the average of four readings.

### 2.6 Muscle biopsy

Biopsy sites were chosen based on MRI findings, and pathology results were interpreted by a pathologist with expertise in myopathies. The evaluation included analysis of muscle fiber necrosis, muscle bundle atrophy, fibrosis, fat infiltration, and fasciitis.

### 2.7 Development and evaluation of predictive models

Two models were developed to assess treatment response in PM/DM patients after conventional therapy: a comprehensive model and a non-invasive model. Univariate and multivariate logistic regression analyses were performed to identify risk factors associated with treatment response. Independent prognostic factors were used to construct clinical predictive models and nomograms. Model performance was evaluated using receiver operating characteristic (ROC) curves and area under the curve (AUC). Calibration curves, generated via bootstrapping (1,000 iterations), assessed the agreement between predicted and actual probabilities. Decision curve analysis (DCA) was applied to evaluate the clinical utility of the models.

### 2.8 Statistical methods

Continuous variables with a normal distribution were reported as mean ± standard deviation (x̄ ± s) and compared using independent samples *t*-tests. Non-normally distributed variables were expressed as medians with interquartile ranges and analyzed with Mann–Whitney U tests. Categorical variables were reported as counts and percentages and compared using χ^2^ tests. Variables with *p* < 0.05 in univariate analysis were included in multivariate logistic regression. All statistical tests were two-sided, with *p* < 0.05 considered statistically significant. Analyses were conducted using R 4.2.1 and the RMS package.

## 3 Results

### 3.1 Characteristics of the study sample

This study enrolled a total of 106 p.m./DM patients, including 64 patients (60.4%) from the Qingdao cohort who underwent muscle biopsy. The Qingdao cohort consisted of 84 patients (53 DM and 31 p.m.), with a female predominance (64.3%, 54/84) and a mean diagnostic age of 57.7 ± 1.5 years. Among these patients, 35.7% (30/84) were classified as non-responders to treatment. The Liaocheng cohort included 22 patients (13 DM and 9 p.m.), also showing a female predominance (68.2%, 15/22) and a mean diagnostic age of 56.4 ± 3.8 years, with 31.8% (7/22) identified as non-responders (Table 1).

**Table 1:** Baseline characteristics for PM/DM patients in two cohorts.

Characteristics	Qingdao cohort			Liaocheng cohort		
	All patients (*n* = 84)	Responders (*n* = 54)	Non-responders (*n* = 30)	All patients (*n* = 22)	Responders (*n* = 15)	Non-responders (*n* = 7)
Gender						
Female	54 (64.3)	40 (74.1)	14 (46.7)	15 (68.2)	12 (80.0)	3 (20.0)
Male	30 (35.7)	14 (25.9)	16 (53.3)	7 (31.8)	3 (42.9)	4 (57.1)
Age, year	57.7 ± 1.5	58.1 ± 2.0	57.0 ± 2.3	56.4 ± 3.8	58.9 ± 4.7	51.1 ± 6.4
Subtypes						
PM	31 (36.9)	23 (42.6)	8 (26.7)	9 (40.9)	7 (46.7)	2 (28.6)
DM	53 (63.1)	31 (57.4)	22 (73.3)	13 (59.1)	8 (53.3)	5 (71.4)
CK (IU/L)	608.6 (128.6–1742.1)	323.3 (87.0–1410.3)	1169.9 (349.0–2129.3)	514.6 (315.7–960.4)	497.0 (171.3–960.4)	532.1 (348.3–1083.4)
MSA						
Anti-Jo-1	15 (17.9)	10 (18.5)	5 (16.7)	3 (13.6)	2 (13.3)	1 (14.3)
Anti-MDA5	3 (3.6)	1 (1.9)	2 (6.7)	1 (4.5)	0 (0)	1 (14.3)
Anti-TIF1γ	7 (8.3)	4 (7.4)	3 (10.0)	2 (9.1)	1 (6.7)	1 (14.3)
Anti-Mi-2	7 (8.3)	4 (7.4)	3 (10.0)	1 (4.5)	1 (6.7)	0 (0)
Anti-NXP-2	4 (4.8)	2 (3.7)	2 (6.7)	1 (4.5)	1 (6.7)	0 (0)
Anti-EJ	8 (9.5)	7 (13.0)	1 (3.3)	2 (9.1)	1 (6.7)	1 (14.3)
MAA						
Anti-PM-Scl	7 (8.3)	3 (5.6)	4 (13.3)	2 (9.1)	1 (6.7)	1 (14.3)
Anti- RNP	6 (7.1)	3 (5.6)	3 (10.0)	2 (9.1)	0 (0)	2 (28.6)
Anti-Ro/SSA	13 (15.5)	9 (16.7)	4 (13.3)	4 (18.2)	3 (20.0)	1 (14.3)
EMG						
Abundant SPs	27 (32.1)	15 (27.8)	12 (40.4)	5 (22.7)	2 (13.3)	3 (42.9)
MRI	5 (3–7)	5 (3–6)	5 (3–7)			
PET-CT						
mSUVmax	0.8 ± 0.03	0.7 ± 0.04	0.9 ± 0.06	0.70 ± 0.06	0.64 ± 0.07	0.82 ± 0.10
Muscle biopsy						
Fiber necrosis	6 (9.4)	2 (4.4)	4 (21.1)			
Bundle atrophy	9 (14.1)	3 (6.7)	6 (31.6)			
Fibrosis	7 (10.9)	2 (4.4)	5 (26.3)			
Fat infiltration	2 (3.1)	1 (2.2)	1 (5.3)			
Fasciitis	1 (1.6)	0 (0)	1 (5.3)			

Data are presented as the mean ± SD, median (interquartile range, IQR), or *n* (%). PM, polymyositis; DM, dermatomyositis; CK, creatine kinase; MSA, myositis-specific autoantibodies; MAA, myositis-associated autoantibodies; EMG, electromyogram; SPs, spontaneous potentials; MRI, magnetic resonance imaging; PET/CT, positron emission tomography/computed tomography; mSUVmax, muscle maximum standardized uptake value.

For model development, 64 p.m./DM patients from the Qingdao cohort with available muscle biopsy results were selected. An additional 20 p.m./DM patients from the same cohort without biopsy results were designated as the internal validation set. The external validation set comprised 22 p.m./DM patients from the Liaocheng cohort.

**Figure 2:**
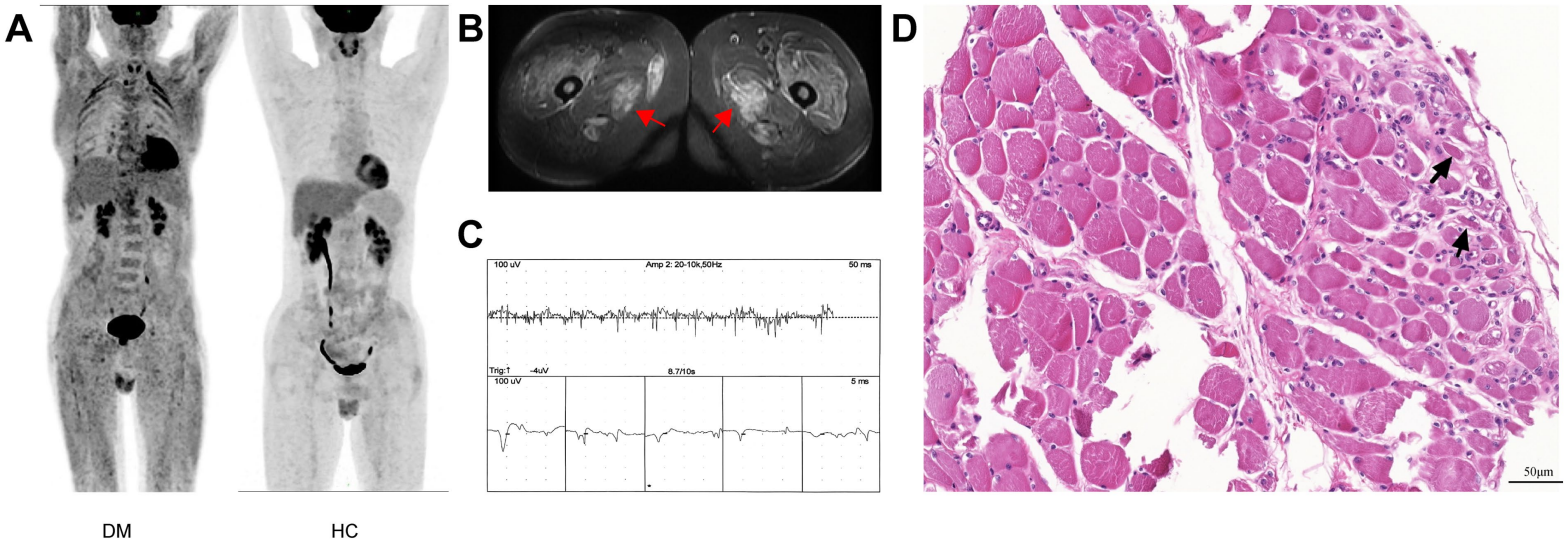
Presentation of results from different diagnostic tools. **(A)** PET-CT: DM patients exhibited increased proximal muscle FDG uptake and bilateral ILD compared to HCs. **(B)** MRI: T2-weighted images with short tau inversion recovery sequence revealed high signal intensity (arrows) in the skeletal muscles of DM patients. **(C)** EMG: EMG of the deltoid muscle in a DM patient showed abundant spontaneous potentials (positive sharp waves). **(D)** Muscle biopsy: Perifascicular muscle bundle atrophy (arrows) was observed in a DM patient (HE stain, ×200). PET-CT, positron emission tomography/computed tomography; DM, dermatomyositis; FDG, fluorodeoxyglucose; ILD, interstitial lung disease; HCs, healthy controls; MRI, magnetic resonance imaging; EMG, electromyography.

In the training set, significant differences were observed between non-responders and responders. Non-responders were predominantly male (68.4% vs. 24.4%, *p* = 0.001) and exhibited higher CK levels (1537.0 [349.0–2306.8] vs. 288.0 [80.0–1467.0], *p* = 0.008). Additionally, non-responders demonstrated a higher frequency of abundant SPs on EMG (52.6% vs. 22.2%, *p* < 0.05). Furthermore, non-responders had significantly higher mSUVmax on PET/CT imaging (1.1 [0.7–1.3] vs. 0.6 [0.5–0.7], *p* < 0.05) and a greater prevalence of muscle fascicular atrophy (31.6% vs. 6.7%, *p* < 0.05) and fibrosis (26.3% vs. 4.4%, *p* < 0.05) on muscle biopsy (Table 2). The characteristics of results from different diagnostic tools in the evaluation of PM/DM are presented in Figure 2.

**Table 2:** Univariate analysis for treatment effect in the study cohort.

Characteristics	Responders (*n* = 45)	Non-responders (*n* = 19)	*p* value	Univariate analysis	*p* value
				OR (95% CI)	
Gender, Male	11 (24.4)	13 (68.4)	0.001	6.70 (2.05–21.85)	0.002
Age, years	59.4 ± 2.2	56.1 ± 2.6	0.381	0.02 (0.98–1.06)	0.376
CK, IU/L	288.0 (80.0–1467.0)	1537.0 (349.0–2306.8)	0.008	1.00 (1.00–1.00)	0.042
Anti-Jo-1	8 (17.8)	3 (15.8)	1.000	0.87 (0.20–3.70)	0.847
Anti-MDA5	1 (2.2)	1 (5.3)	0.509	2.44 (0.15–41.24)	0.535
Anti-TIF1γ	2 (4.4)	3 (15.8)	0.150	4.03 (0.62–26.39)	0.146
Anti-Mi-2	3 (6.7)	2 (10.5)	0.629	1.65 (0.25–10.75)	0.602
Anti-SAE	1 (2.2)	1 (5.3)	0.509	2.44 (0.15–41.24)	0.535
Anti-NXP-2	1 (2.2)	2 (10.5)	0.208	5.18 (0.44–60.88)	0.191
Anti-EJ	6 (13.3)	1 (5.3)	0.664	0.36 (0.04–3.23)	0.362
Anti-PM-Scl	3 (6.7)	1 (5.3)	1.000	0.78 (0.08–8.00)	0.833
Anti- RNP	3 (6.7)	2 (10.5)	0.629	1.65 (0.25–10.75)	0.602
Anti-Ro/SSA	6 (13.3)	4 (21.1)	0.466	1.73 (0.43–7.02)	0.441
EMG, Abundant SPs	10 (22.2)	10 (52.6)	0.016	3.89 (1.24–12.19)	0.020
MRI	5 (3–6)	5 (2–7)	0.935	0.99 (080–1.23)	0.941
mSUVmax	0.6 (0.5–0.7)	1.1 (0.7–1.3)	<0.001	0.02 (0.00–0.17)	<0.001
Fiber necrosis	2 (4.4)	4 (21.1)	0.059	5.73 (0.95–34.56)	0.057
Bundle atrophy	3 (6.7)	6 (31.6)	0.016	6.46 (1.41–29.52)	0.016
Fibrosis	2 (4.4)	5 (26.3)	0.021	7.68 (1.34–44.06)	0.022
Fat infiltration	1 (2.2)	1 (5.3)	0.509		
Fasciitis	0 (0)	1 (5.3)	0.297		

Data are presented as the mean ± SD, median (interquartile range, IQR), or *n* (%). OR, odds ratio; 95% CI, 95% confidence interval; CK, creatine kinase; EMG, electromyogram; SPs, spontaneous potentials; MRI, magnetic resonance imaging; PET/CT, positron emission tomography/computed tomography; mSUVmax, muscle maximum standardized uptake value; *Statistically significant and participated in model construction.

### 3.2 Model building

**Figure 3:**
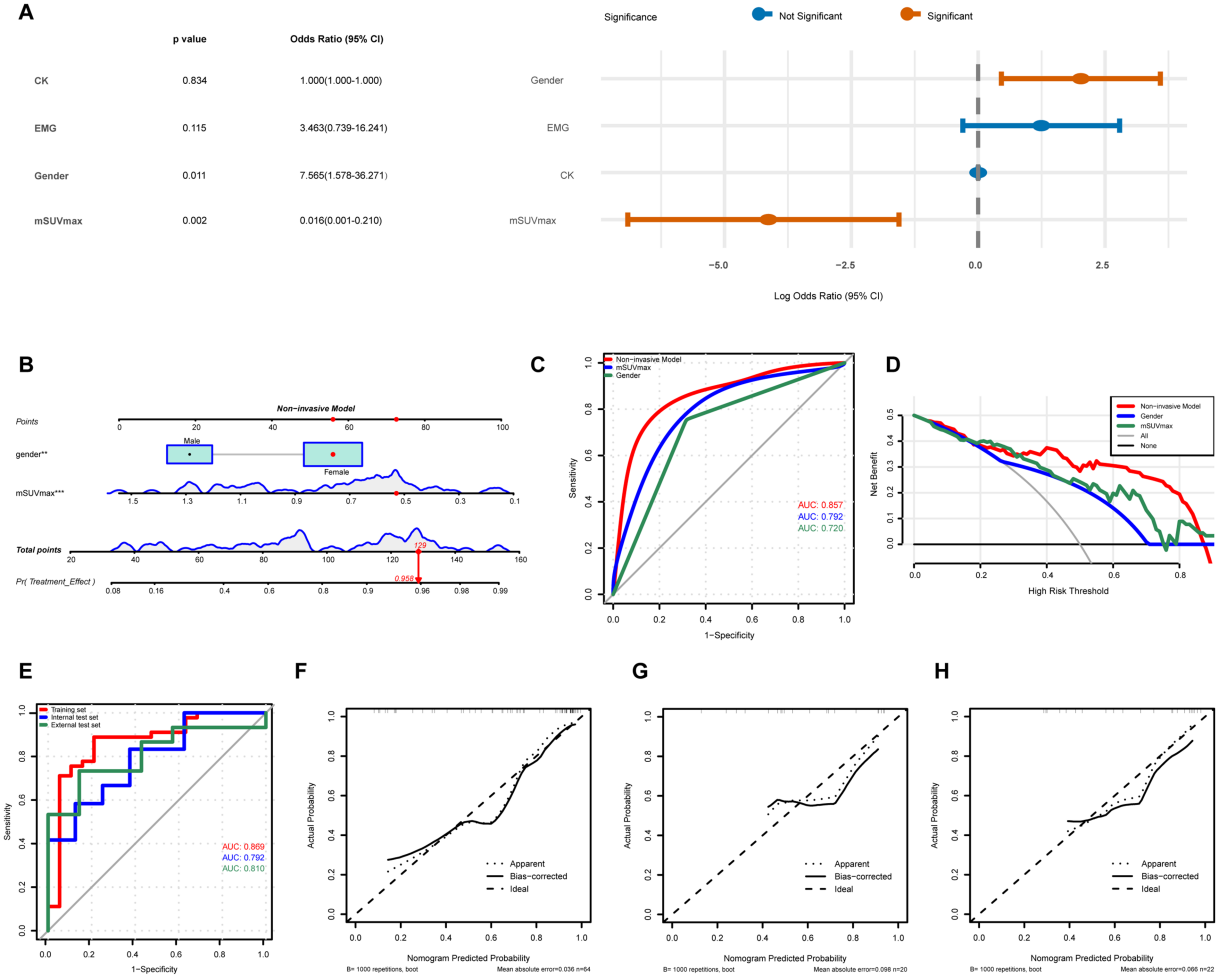
Construction and validation of the non-invasive nomogram predictive model. **(A)** The forest graph shows the results of multivariate logistic regression analysis without muscle biopsy diagnosis. **(B)** Nomogram of the non-invasive model for predicting non-response to conventional treatment, integrating factors such as gender and mSUVmax. **(C)** ROC curves comparing the performance of individual factors (gender and mSUVmax) with that of the non-invasive model. **(D)** DCA curves to evaluate the clinical utility of the non-invasive model for predicting response to conventional treatment. **(E)** ROC curves for the discrimination of the nomogram. Calibration curves for non-invasive model in the **(F)** training set (*n* = 64), **(G)** internal test set (*n* = 20), and **(H)** external test set (*n* = 22). 95% CI, 95% confidence interval; CK, creatine kinase; EMG, electromyogram; mSUVmax, muscle maximum standardized uptake value; ROC, receiver operating characteristic; DCA, decision curve analysis; AUC, area under the curve.

Univariate (Table 2) and multivariate (Table 3) logistic regression analyses were performed on clinical data from the model development cohort. The results identified male sex (OR 13.94, 95% confidence intervals [CI]: 1.861–104.467, *p* = 0.011), PET/CT mSUVmax (OR 0.01, 95% CI: 0.000–0.207, *p* = 0.004), and muscle fascicular atrophy (OR 24.52, 95% CI: 1.64–366.0, *p* = 0.020) as independent predictors of non-response to conventional treatment. After excluding muscle biopsy results, the independent risk factors for non-response were male sex (OR 7.565, 95% CI: 1.578–36.271, *p* = 0.011) and PET/CT mSUVmax (OR 0.016, 95% CI: 0.001–0.210, *p* = 0.002) (Figure 3A).

**Table 3:** Multivariate analysis for treatment effect in the study cohort.

Characteristics	Multivariate analysis	*p* value
	OR (95% CI)	
Gender, Male	13.94 (1.86–104.47)	0.011*
CK, IU/L	1.00 (1.00–1.00)	0.950
EMG, Abundant SPs	1.06 (0.14–8.29)	0.957
mSUVmax	0.01 (0.00–0.207)	0.004*
Bundle atrophy	24.52 (1.64–366.00)	0.020*
Fibrosis	13.81 (0.692–275.89)	0.066

Data are presented as the mean ± SD, median (interquartile range, IQR), or *n* (%). OR, odds ratio; 95% CI, 95% confidence interval; CK, creatine kinase; EMG, electromyogram; SPs, spontaneous potentials; mSUVmax, muscle maximum standardized uptake value; *Statistically significant and participated in model construction.

Independent predictors identified through multivariate logistic regression analysis were used to construct a nomogram prediction model. Two models were developed: a comprehensive model (male sex, mSUVmax, and muscle fascicular atrophy) and a non-invasive model (male sex and mSUVmax), with corresponding nomograms created for each.

### 3.3 Evaluation and validation of model performance

ROC curves and DCA were used to evaluate the predictive performance of independent factors for non-response to conventional treatment and their clinical utility.

**Figure 4:**
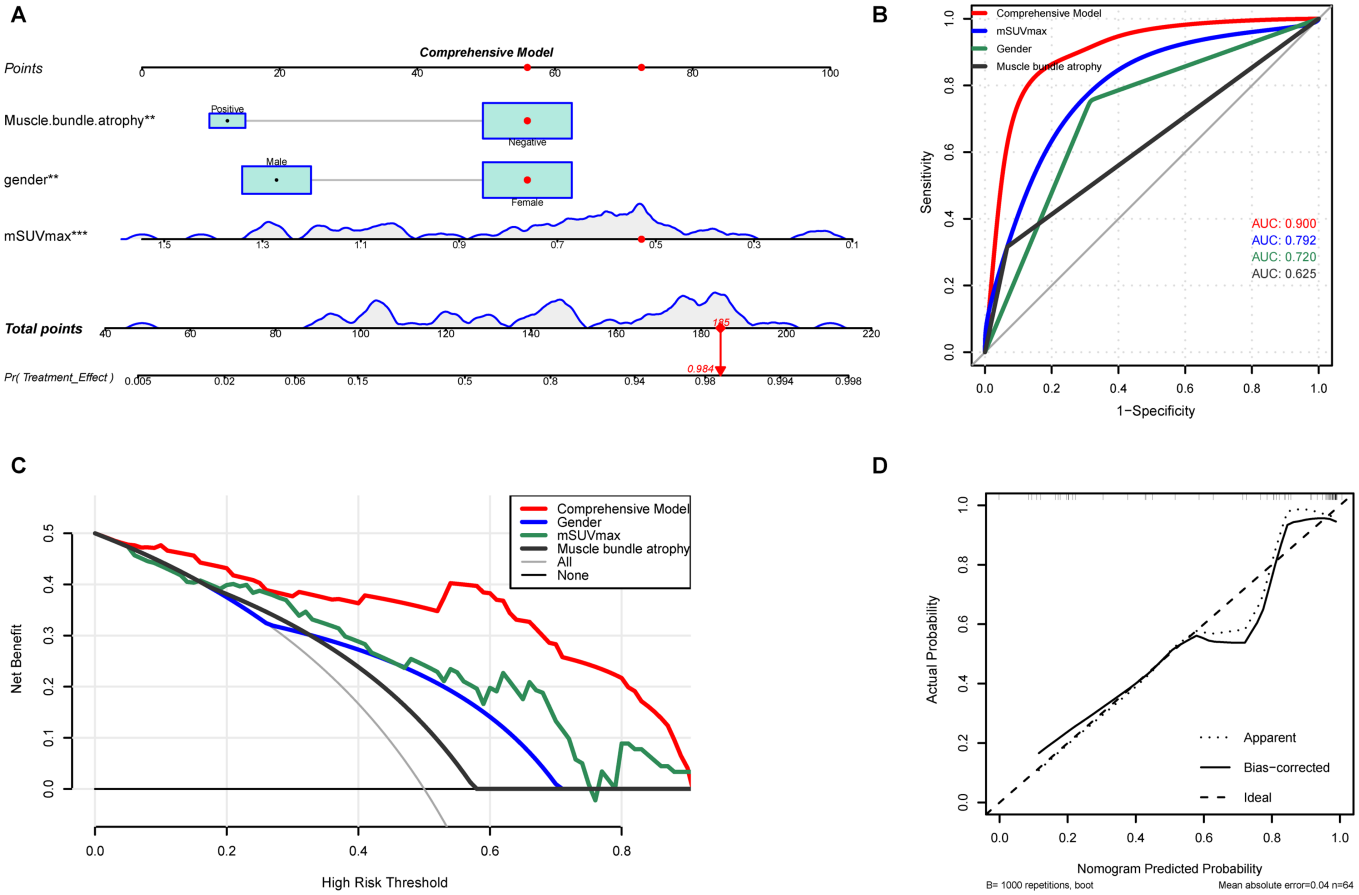
Construction and validation of the comprehensive nomogram predictive model. **(A)** Nomogram of the comprehensive model for predicting non-response to routine treatment, integrating factors such as gender, muscle bundle atrophy, and mSUVmax. **(B)** ROC curves comparing the performance of individual factors (gender, muscle bundle atrophy, mSUVmax) with that of the comprehensive model. **(C)** DCA of the nomogram, showing the net benefit of using the nomogram-based predictions at various threshold probabilities for clinical decision-making. **(D)** Calibration curve for the comprehensive model, comparing predicted vs. observed probabilities of non-response to treatment. ROC, receiver operating characteristic; mSUVmax, muscle maximum standardized uptake value; DCA, decision curve analysis.

The nomogram for the comprehensive model was shown in Figure 4A. The comprehensive model achieved the highest AUC of 0.900 (95% CI: 0.813–1.009), surpassing individual predictors (Figure 4B). Across the 0–90% threshold probability range, the comprehensive model provided a greater net benefit than single-factor models (Figure 4C). Calibration curves demonstrated a strong agreement between predicted and observed outcomes (Figure 4D).

The nomogram for the non-invasive model was shown in Figure 3B. The non-invasive model achieved a higher AUC than individual predictors (Figure 3C), with values of 0.869 (95% CI: 0.766–0.972) in the training set, 0.792 (95% CI: 0.599–0.984) in the internal test set, and 0.810 (95% CI: 0.632–0.987) in the external test set (Figure 3E). Within a reasonable threshold probability range, the non-invasive model demonstrated a greater net benefit than single-factor models (Figure 3D). Model performance was further assessed through internal and external validation. Bootstrap analysis with 1,000 resamples showed good agreement between predicted and observed probabilities. The Hosmer-Lemeshow test indicated no significant difference between predicted and observed outcomes, confirming the model’s calibration accuracy (Figures 3F–H).

## 4 Discussion

IIM present with diverse clinical manifestations and significant variability in therapeutic responses. This study conducted a comprehensive evaluation of multiple diagnostic modalities and, for the first time, developed two distinct predictive models to address different clinical requirements. The comprehensive model, which integrates clinical, pathological, and imaging features of PM/DM patients, effectively identified individuals who are non-responsive to conventional treatments. The non-invasive model, designed for broader applicability, demonstrated high predictive accuracy across the training set, internal validation set, and external validation set.

These models enable a paradigm shift from a reactive to a proactive treatment strategy. The conventional approach is reactive, waiting 3 months to declare treatment failure, which leads to prolonged morbidity and increased tissue damage. In contrast, by predicting non-response at baseline, our models can potentially shorten the time to effective therapy for the most difficult-to-treat patients. This aligns with the core goals of personalized medicine: to deliver the right drug to the right patient at the right time.

Demonstrating the practical utility of these predictive models is paramount. The non-invasive model, relying on readily available variables, serves as an efficient initial screening tool, enabling early risk assessment at diagnosis without awaiting biopsy results. For high-risk patients identified by the non-invasive model or those with diagnostic uncertainty, the comprehensive model, which incorporates muscle biopsy data, provides higher certainty in stratifying the highest-risk individuals, thereby justifying more aggressive or alternative treatment strategies from the outset. Both models facilitated the identification of patients unresponsive to conventional therapies, enabling stratified management strategies at initial diagnosis. By providing robust support for clinical decision-making, these models hold significant promise for improving patient outcomes. For patients identified as high-risk for non-response, evidence-based treatment modifications can be initiated immediately (11): (1) First-line intensification with combination immunosuppressive therapy from the outset; (2) Early escalation to biologics (e.g., Rituximab or IVIG) for the highest-risk cases; and (3) Enhanced monitoring with closer clinical and laboratory follow-up to detect suboptimal response early. This proactive approach aims to circumvent treatment failure without awaiting the conventional 12-week assessment.

In this study, 106 p.m./DM patients were included, with 34.9% (37/106) exhibiting inadequate responses to a 12-week regimen of conventional treatment involving corticosteroids and immunosuppressants, consistent with previous reports (7, 14). Logistic regression analysis identified male sex, muscle fascicular atrophy, and mSUVmax as independent risk factors for poor treatment response, highlighting their predictive value. Notably, research on treatment response predictors in PM/DM remains limited, often relying on small, single-center studies, clinical trials, or case reports, with insufficient consensus to guide individualized therapy. However, prior studies focusing on mortality have identified adverse prognostic factors, such as advanced age (>60 years), male sex, fever, anti-MDA5 antibody positivity, elevated ferritin levels, C-reactive protein >1 mg/dL, and oxygen saturation <95% (15, 16). Our findings corroborate these results, revealing a higher proportion of males among non-responders (*p* < 0.05). The identification of male sex as a risk factor for non-response is particularly intriguing, especially given the generally higher susceptibility of females to autoimmune diseases. While the strength of this association (OR>10) may be influenced by sample size and cohort characteristics, it is consistent with broader epidemiological evidence. Multiple studies have independently identified male sex as a significant predictor of poorer overall prognosis in IIM, including association with higher mortality (17, 18). Although our endpoint (short-term treatment non-response) differs from mortality, both likely share common underlying drivers, such as a more aggressive disease phenotype or intrinsically reduced responsiveness to immunosuppressive therapy. This established context provides biological and clinical plausibility to our observation. Furthermore, certain sociobehavioral and comorbidity factors, such as differences in comorbidity distributions or health-seeking behaviors, may indirectly influence treatment outcomes. Thus, for patients predicted to have poor treatment responses, initiating more aggressive therapeutic strategies at diagnosis may be a prudent approach.

PM/DM is characterized by inflammatory lesions in striated muscles. Recent advances in imaging techniques have significantly impacted the diagnosis and evaluation of disease activity in PM/DM. Studies have demonstrated a strong correlation between PET/CT findings and muscle abnormalities detected by EMG or MRI, with PET/CT showing superior sensitivity in detecting muscle inflammation (19, 20). PET/CT not only distinguishes PM/DM from non-myopathic conditions but also reflects disease activity through increased FDG uptake in affected muscles, which can guide muscle biopsy site selection and serve as a marker for myopathy activity (21, 22). Furthermore, elevated SUV ratios between proximal muscles and the longissimus thoracis are associated with disease severity, with ratios exceeding 1.73 indicative of active DM (23). In this study, proximal muscle SUVmax analysis revealed significantly higher values in non-responders compared to responders to conventional treatment, suggesting that elevated SUVmax may reflect active or more severe disease. These findings underscore the potential of PET/CT as a diagnostic tool to predict outcomes of PM/DM treatment.

Muscle biopsy remains essential for PM/DM diagnosis. Approximately 40% of IIM patients are MSA-negative, and muscle pathology aids in diagnosis, achieving a diagnostic yield of approximately 50% (24, 25). Muscle biopsy findings further aid in subtype classification based on distinct pathological features. Bundle and perifascicular atrophy are characteristic of DM (26). DM has a worse long-term prognosis than other IIM subtypes, due to higher rates of ILD and malignancies (27). This study found that non-responders to conventional treatment had a higher prevalence of the DM subtype and more frequent bundle atrophy. These results suggest that DM patients, especially those with muscle bundle atrophy, might require more aggressive therapeutic strategies at the onset of treatment compared to PM patients. This study underscores the importance of muscle biopsy for predicting the prognosis of PM/DM.

Although muscle biopsy is a well-established technique with good tolerance and a low risk of postoperative complications, its use remains a subject of debate among experts (28). For DM patients with typical clinical features and positive MSA, diagnostic muscle biopsy may not be deemed necessary for further confirmation (29). Furthermore, as an invasive procedure, muscle biopsy is often less accessible in primary healthcare settings due to limitations in trained personnel and pathology resources. Patient compliance can also be limited by concerns about potential risks, further restricting the use of this diagnostic tool. In this study, 60.4% of patients in the Qingdao cohort underwent muscle biopsy, whereas none in the Liaocheng cohort underwent the procedure. To address the diverse needs of different clinical settings, two predictive models were developed: a comprehensive model and a non-invasive model. The comprehensive model integrated three independent risk factors: sex, muscle biopsy findings, and PET/CT mSUVmax. The non-invasive model excluded muscle biopsy findings, rendering it more applicable across varied diagnostic and treatment environments.

During the validation phase, the model demonstrated generalizability in both internal and external validation sets. To assess its stability, 1,000 iterations of bootstrapping cross-validation were performed. In the external validation set of the non-invasive model, a reduction in the AUC and a widening of the 95% CI were observed compared to the training set, suggesting potential limitations in model stability. These limitations, inherent to multicenter studies, may arise from factors such as the low incidence of PM/DM leading to restricted sample sizes, variability in PET/CT scanning equipment across centers, and inconsistencies in subjective self-assessment criteria for clinical response. Despite these challenges, the model maintains considerable clinical value in its current external application context.

This study has several limitations. First, as a retrospective investigation, it was susceptible to unavoidable selection bias, which may affect the generalizability and accuracy of the findings. Second, the low prevalence of PM/DM resulted in a limited sample size for model development, with validation conducted in only one external center, raising concerns about potential overfitting. The grouping of DM and PM patients may overlook important pathophysiological and clinical differences between these subtypes, and that heterogeneity within DM (particularly driven by MSA status) represents a significant source of prognostic variability that our current model does not capture. Therefore, Future studies with larger, prospective cohorts are warranted to validate our findings and to explore the development of subtype-specific (e.g., DM vs. PM) or MSA-stratified prediction models, which may provide even greater prognostic precision. Third, the study did not comprehensively account for other factors, such as lifestyle and comorbidities, that could influence PM/DM prognosis. Finally, although PM/DM patients face a heightened risk of malignancies within 3 years of diagnosis, this study evaluated treatment response only after 12 weeks of conventional therapy, lacking long-term follow-up data. Future studies with larger, prospective cohorts are warranted to validate our findings and to explore the development of subtype-specific (e.g., DM vs. PM) or MSA-stratified prediction models, which may provide even greater prognostic precision.

## 5 Conclusion

The two predictive models, integrating PET/CT, muscle biopsy, and clinical factors, demonstrated strong predictive capability for identifying treatment-refractory patients unresponsive to conventional therapy. By leveraging these models, clinicians can better tailor therapeutic approaches, potentially improving outcomes for PM/DM patients. The integration of advanced imaging techniques and comprehensive clinical data represents a significant step forward in the personalized management of this complex disease.

## Data Availability

The raw data supporting the conclusions of this article will be made available by the authors, without undue reservation.
